# Supplementary material for: Multimodal Optical Imaging and Modulation with Simultaneous Electrophysiology Through Smart Dura in Non‐Human Primates
Source: Adv Sci (Weinh). 2026 Jan 12;13(25):e14419. doi: 10.1002/advs.202514419 (PMC13137837; doi:10.1002/advs.202514419)
Supplement: Supplementary file 1 — Supporting Information [file ADVS-13-e14419-s002.docx]

**[Supporting Information]**

**Multimodal Optical Imaging and Modulation with Simultaneous Electrophysiology through Smart Dura in Non-Human Primates**

*Nari Hong^1,2^, Sergio Montalvo Vargo^3^, Gaku Hatanaka^2,4^, Zhaoyu Gong^1^, Noah Stanis^1,2^, Jasmine Zhou^1,2^, Tiphaine Belloir^1,2^, Ruikang K. Wang^1^, Wyeth Bair^2,4^, Maysamreza Chamanzar^3,5,6*^, Azadeh Yazdan-Shahmorad^1,2,7,8*^*

1. Department of Bioengineering, University of Washington, Seattle, WA, 98195, USA.

2. Washington National Primate Research Center, Seattle, WA, 98195, USA.

3. Department of Electrical and Computer Engineering, Carnegie Mellon University, Pittsburgh, PA, 15213, USA.

4. Department of Neurobiology and Biophysics, University of Washington, Seattle, WA, 98195, USA.

5. Department of Biomedical Engineering, Carnegie Mellon University, Pittsburgh, PA, 15213, USA.

6. Carnegie Mellon Neuroscience Institute, Pittsburgh, PA, 15213, USA.

7. Department of Electrical and Computer Engineering, University of Washington, Seattle, WA, 98195, USA.

8. Weill Neurohub

*Correspondence: mchamanzar@cmu.edu and azadehy@uw.edu


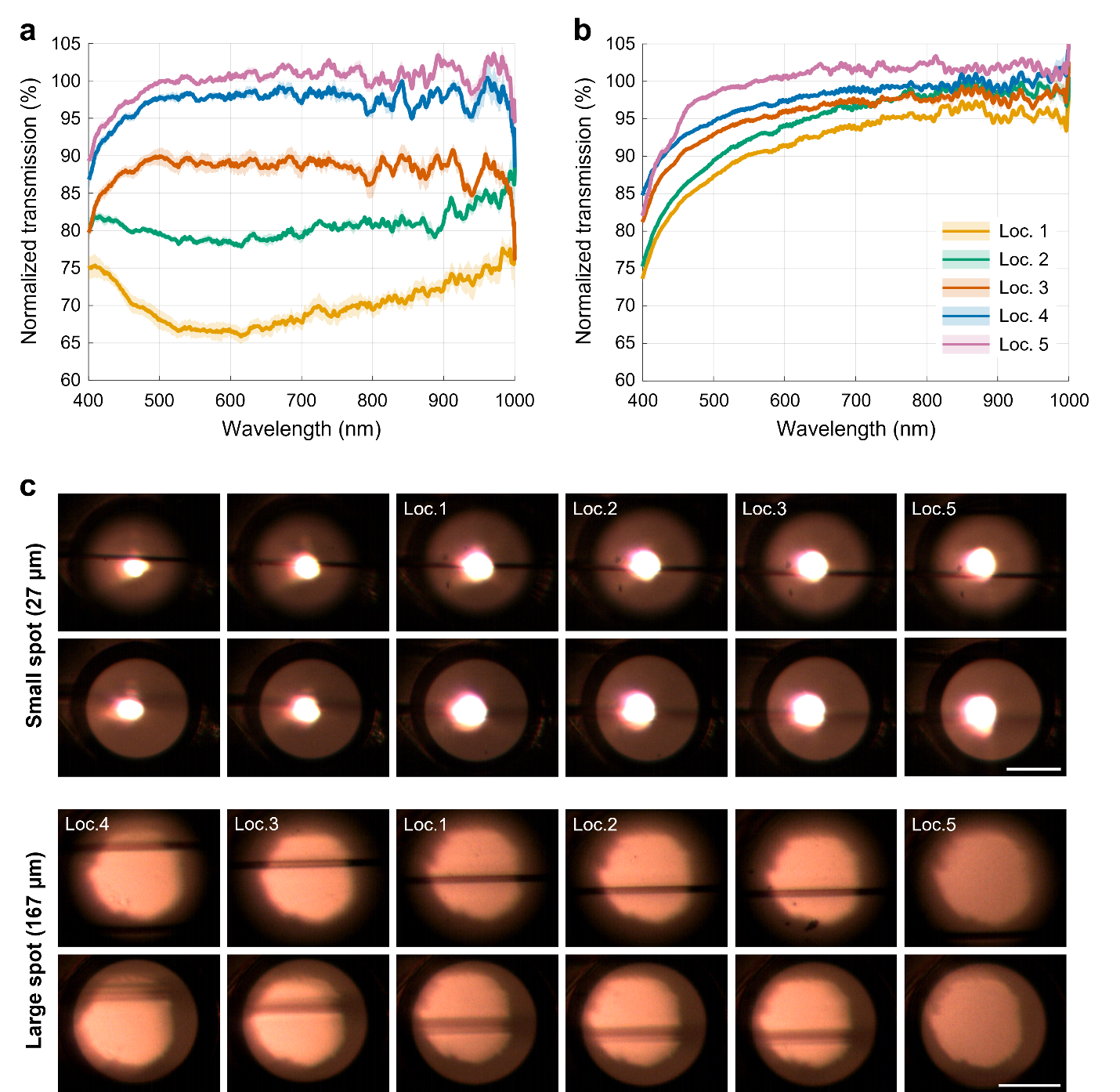


**Figure S1. Transmission measurements through Smart Dura with different light blockage levels.** (a) Transmission spectra for a 27 μm diameter spot at five locations of a 10 μm-wide metal trace with different light blockage levels. Loc. 1 and Loc. 5 are the locations with the highest and lowest levels of the blockage, respectively. Data are normalized to the average value of the least blockage (Loc. 5) and presented as the mean (solid line) ± standard deviation (shaded area). n = 5 measurements. (b) Transmission spectra for a 167 μm diameter spot at five locations of a 10 μm-wide metal trace with different light blockage levels. (c) 1D-scan transmission measurement for two different spot sizes. For each spot size, the top and bottom rows show images focused on the metal trace and an optical fiber, respectively. The exposure time was set long enough to saturate the images to show the metal traces and light spots. Scale bars: 100 μm.


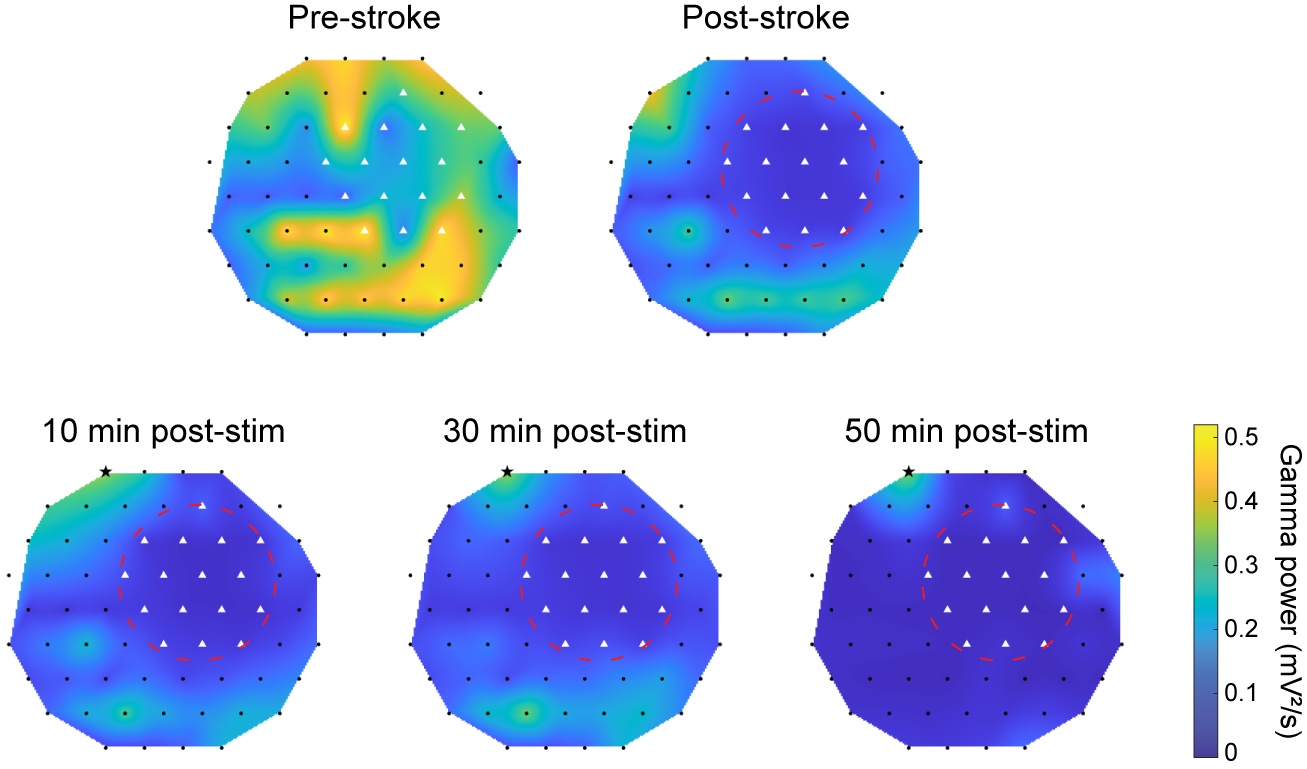


**Figure S2. Heatmaps of gamma power in pre-stroke, post-stroke, and post-electrical stimulation periods.** A red dashed circle and white triangles denote the stroke-induced region and the electrodes within the area, respectively.


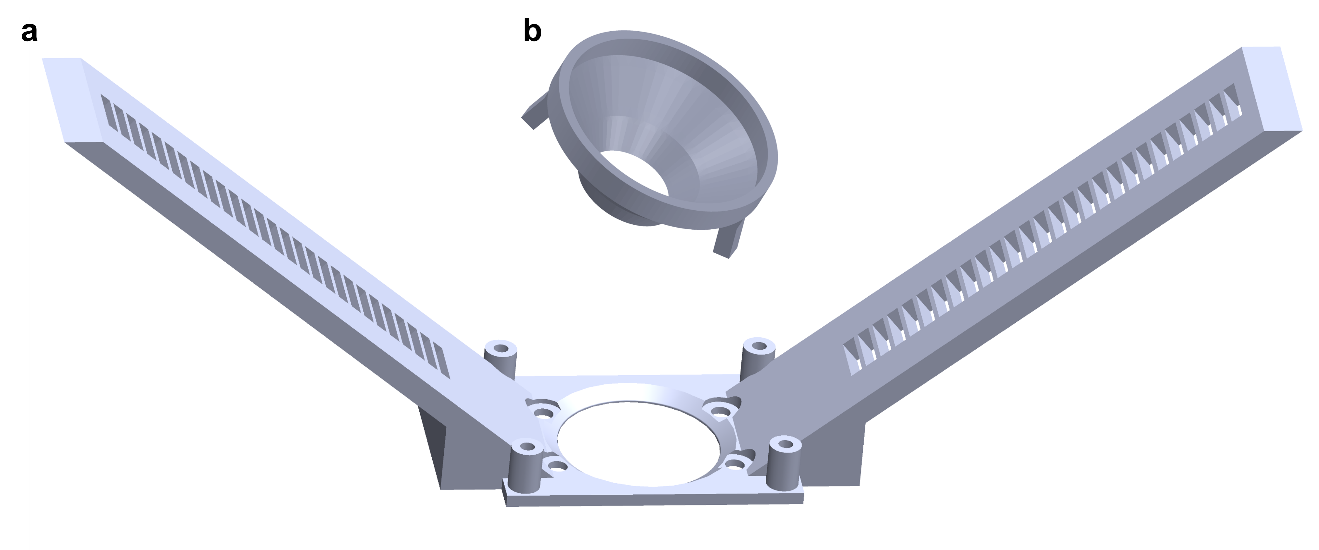


**Figure S3. Designs for custom 3D-printed parts.** (a) A custom holder used to support the two connecting arms of the Smart Dura and the attached recording components. (b) A custom imaging well with an 18 mm diameter glass bottom used to immerse the objective lens in DI water and flatten the target area.


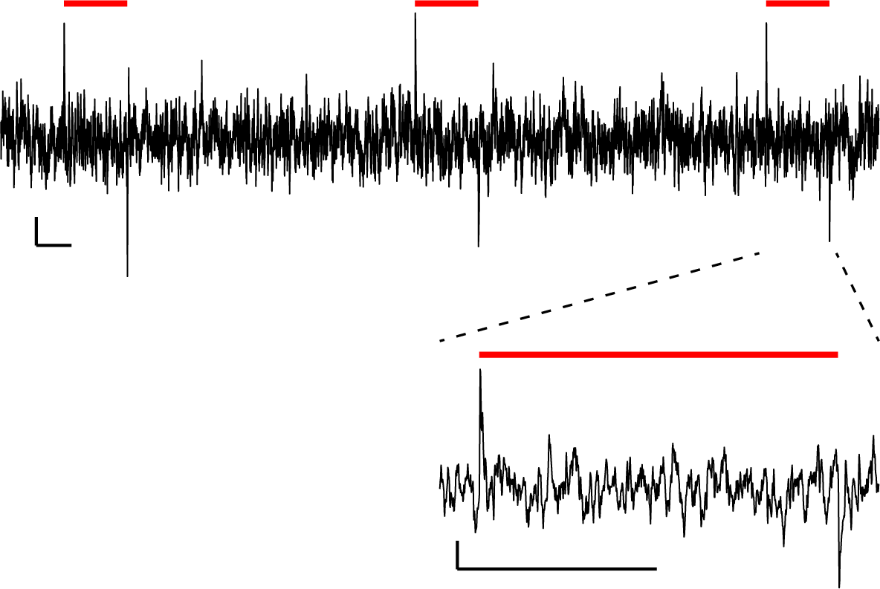


**Figure S4. Photoelectrical artifact generated by light pulses.** A representative low-pass filtered signal trace measured in saline while delivering light stimulation. Red lines indicate the stimulation pulses. Scale bars: 0.5 s and 20 μV.
